# Advanced Nanomaterials and Energetic Application: Experiment and Simulation

**DOI:** 10.3390/nano16020137

**Published:** 2026-01-20

**Authors:** Weiqiang Pang, Djalal Trache, Kaili Zhang

**Affiliations:** 1 National Key Laboratory of Energetic Materials, Xi’an Modern Chemistry Research Institute, Xi’an 710065, China; 2 Teaching and Research Unit of Energetic Processes, Ecole Militaire Polytechnique, Algiers 16046, Algeria; djalaltrache@gmail.com; 3 Department of Mechanical Engineering, City University of Hong Kong, Hong Kong, China

In recent years, significant advancements have been made in the exploitation, combustion, ignition, and application of innovative nano-metric energetic materials (nEMs), including solid fuels, energetic combustion catalysts, metal particles, thermites, energetic composites, and more, thanks to new technological developments in the field of nano-scale science and technology. Utilizing innovative nEMs and their composites in various chemical propulsion systems is beneficial because of their high heat of formation, high specific surface area, high reactive activation, and high energy density. However, although the extremely high heat release rates and tailored burning rates of innovative nEMs are attractive features, their production of high energy in their combustion processes and high combustion efficiency in industry applications presents a great challenge to many engineers and scientists. Various techniques have recently been developed to overcome these intrinsic difficulties. Fundamental research investigations have also been conducted to explore the detailed physicochemical processes associated with the preparation, combustion, and application of innovative nEMs. In particular, state-of-the-art rocket propulsion systems have benefited greatly from the development of innovative nEMs in recent years, especially in terms of prospective nEMs for future rocket fuels and the fabrication of propellants, explosives, and pyrotechnics.

This Special Issue presents the compiled results of the most recent developments of innovative nEMs, which include nano-sized metal fuels, nano-sized combustion catalysts, nano-sized energetic composite nano-sized oxidizers, nano-sized thermites, and more, to be used in simulations, ignition, and combustion, with a particular focus on energetic application technology in chemical propulsion systems. This effort focuses on the design and investigation of novel high-tech nEMs for metal fuels, oxidizers, combustion catalysts, thermites, and further additives for solid, liquid, gelled, hybrid, electro-controlled, and propellant systems. On the one hand, considerable effort is exerted to improve and perfect the propulsion systems themselves, which are designed exclusively for the nano-metric ingredients that they work with. On the other hand, researching new nano-metric ingredients for chemical propulsion represents a challenge. In recent years, in the realm of nano-metric energetic material ingredients for chemical propellants, great progress has been made in the development of propellants, explosives, and pyrotechnics. Yet, despite the impressive progress witnessed in the field of nano-EMs during the last century, it must be admitted that the rate of progress is much slower than in other fields, such as polymer chemistry, electronics, and computers, due to the various constraints and restrictions that nano-EM scientists encounter when developing a new nEM. These include safety, stability (thermal, storage, etc.), cost, and other considerations.

To accelerate the potential applications, various works have focused on the physical and chemical characteristics through theory, experiments, and simulations. The aim of this issue is to present a comprehensive knowledge on the synthesis, characterization, combustion, mechanical, detonation, and safety of nEMs. This Special Issue ***Advanced Nanomaterials and Energetic Application: Experiment and Simulation*** explores innovative nEMs and nEM ingredients, as well as the experiment and simulation of formulations. It collects contributions spanning the recent progress and data of nEMs in chemical propulsion and applications. Attention has been paid to the design, model, properties, and state-of-the-art nature of this class of thermochemical propulsion devices. A total of 15 papers were selected for publication, following a standard peer review process, which represent the most recent achievements of famous research groups. The participation of young authors with novel or innovative concepts was particularly encouraged, and they were of course supported by the assistance of their supervisors.

Aluminum powder is the most commonly used metal fuel in the explosives industry, and is widely used in explosives and propellants. The application of aluminum powder in explosives can greatly improve the detonation heat and work power, as well as the damage efficiency of the ammunition. Aluminum-containing explosives are widely used in ammunition for air defense weapons, ground targets, and underwater weapons. The application of aluminum powder in propellants can significantly increase their combustion heat and improve the specific impulse of rocket engines.

The research progress in the preparation technology, reactivity, and application of nano-aluminum in explosives and propellants is systematically reviewed here, and the reactivity differences between nano-aluminum powder and micro-aluminum powder are compared [1]. It has been established that nano-sized aluminum exhibits a significantly higher reactivity than its micron-sized counterpart, as evidenced by its lower ignition threshold, enhanced combustion behavior, and greater reaction completeness. These characteristics result in a more pronounced impact both on the detonation performance of mixed explosives and on the combustion efficiency of propellant formulations.

As the global energy transition accelerates and the integration of renewable energy into power grids presents new challenges, the development of electrochemical energy storage materials which are capable of delivering both high power density and high energy density has become a priority in energy research. Since the emergence of two-dimensional transition metal carbides and nitrides (MXenes), these materials have been of great interest due to their tunable interlayer spacing, excellent hydrophilicity, high electrical conductivity, compositional versatility, and chemically rich surface characteristics. More than 100 different MXene combinations can be calculated theoretically, but only more than 40 have been successfully synthesized through experiments. Among the many synthesized and reported MXene materials, vanadium-based carbide MXenes, represented by V_2_CT*_x_* and V_4_C_3_T*_x_*, show excellent application prospects in energy storage and have become a key focus for researchers. In this review, the structure, characteristics, and preparation methods of vanadium-based MXene precursors in the MAX phase, and their applications in supercapacitors, are discussed. The key challenges currently associated with vanadium-based materials and their heterostructures are highlighted, and an outline of the promising future research directions is presented [2].

Metal and metalloid powders are widely used in high-energy compositions (HECs) and solid propellants (SPs), with the effect of increasing their energetic characteristics in the combustion chamber. The particle size distribution, protective coatings of the particles, and heat of combustion of the metal powders influence both the ignition and combustion parameters of the HECs, as well as the characteristics of the propulsion systems [3]. The effect of Al-B, Fe-B, and Ti-B (Me-B) mixture ultrafine powders (UFPs) on the ignition and combustion characteristics of a model HEC, which is based on a solid oxidizer and a polymer combustible binder, was investigated. The Me-B mass ratios in the mixture UFPs corresponded to the phase composition of the borides AlB_2_, FeB, and TiB_2_. It was found that by replacing the aluminum UFP with Al-B, Fe-B, and Ti-B UFPs in the HECs, the exponent (*n*) in the correlations of the ignition delay time *t*_ign_(*q*) and burning rate *u*(*p*) changed. The maximum burning rate and *n* over the pressure range of 0.5–5.0 MPa were obtained for the HEC with Al-B UFPs due to the increase in the heat release rate near the sample surface during the joint combustion of the Al and B particles.

Aluminum (Al) powder is a metal fuel used uniquitously in solid propellants, and its practical energy release significantly impacts the performance of solid propellants. The enhanced energy release is a consequence of the increased energy in Al powder and its improved combustion efficiency. To enhance the energy release of Al powder in solid propellants, poly (difluoroaminomethyl-3-methylethoxybutane) (PDF), which has difluoroamino (NF_2_), was utilized to improve the energy and promote combustion efficiency. Al with three distinct powder sizes (29 μm, 13 μm, and 1~3 μm) was coated with PDF using the solvent/non-solvent method, which led to the formation of Al/PDF composites. The morphology and characteristics of this Al/PDF were characterized [4], and the results demonstrated that all powder sizes of Al/PDF had core–shell structures, and that the NF_2_ in the PDF layer on the Al surface maintained its original structure. Al/PDF exhibited greater hydrophobicity. NF_2_ prompted Al/PDF, with better catalysis upon ammonium perchlorate (AP) decomposition. Compared to the Al powder, the ignition delay time of the Al/PDF was significantly reduced. For the mixed samples of Al/PDF and AP, NF_2_ shortened the ignition delay time, improved the combustion stability, extended the combustion duration, and formed volatile fluorine compounds. These findings underscore the effect of NF_2_ in Al/PDF composites, which enhance the energy release of Al and offer promising potential applications.

Due to the increase in the application of antibacterial nanomaterials (NMs), the potential for their ingestion by humans has also increased. Evidence shows that NMs can induce dysbiosis in the gut microbiota in vivo. Despite this, in vitro investigation of the antibacterial activity of NMs on gut-relevant, commensal bacteria has been neglected, with studies predominantly assessing NM toxicity against pathogenic bacteria. Thus, the current study investigates the antibacterial activity of copper oxide (CuO) NMs to Escherichia coli K12, Enterococcus faecalis, and Lactobacillus casei, using a combination of approaches to evaluate the importance of reactive oxygen species (ROS) production as a mechanism of toxicity [5]. The data suggest that CuO NMs have antibacterial activity against gut-relevant bacteria, with evidence that NM-mediated ROS production may contribute to reductions in bacterial viability, which suggests that the use of a combination of assays provides a robust assessment of the antibacterial properties of ingested NMs. In particular, it is recommended that plate counts and OD measurements be prioritized in future screenings of the antibacterial properties of NMs.

Progress and innovation in material science increasingly rely on the development of next-generation materials that integrate multi-functionality, sustainability, and responsiveness to external stimuli, like heat, light, or mechanical stress. These features are essential to meeting the escalating demands for high-performance materials in advanced applications, including flexible electronics, advanced sensing technologies, and smart integrated platforms. Composite films based on phosphorylated polyvinyl alcohol (PVA-P), Ti_3_C_2_T_x_ MXene, and cholesteryl acetate (ChLC) were designed and characterized to explore their potential in flexible electronic applications [6]. The incorporation of phosphate groups and ChLC in these films enhanced the intermolecular interactions, which was confirmed by FTIR spectroscopy. Morphological and optical analyses revealed a transition from homogeneous to phase-separated structures with birefringent textures in ChLC-rich films. Thermal studies demonstrated their improved stability and increased glass transition and melting temperatures, particularly in samples with higher ChLC contents. Mechanical and dielectric evaluations, meanwhile, highlighted the tunability of the stiffness, flexibility, permittivity, and dielectric losses depending on the MXene and ChLC ratios. These multifunctional films exhibit flame-retardant behavior and show promise for use in stimuli-responsive, sustainable electronic devices, such as flexible displays and sensors.

Vaterite particles, naturally occurring mineral forms of polycrystalline calcium carbonate (CaCO_3_), are both biocompatible and biodegradable, making them promising candidates for targeted drug delivery. Biocompatible vaterite microspheres, which are renowned for their porous structure, are promising carriers for magnetic nanoparticles (MNPs) in biomedical applications such as targeted drug delivery and diagnostic imaging. Having precise control over the magnetic moment of individual microspheres is crucial for these applications. This study employs wide-field quantum diamond microscopy to map the stray magnetic fields of individual vaterite microspheres (3–10 μm) which are loaded with Fe_3_O_4_ MNPs of varying sizes (5 nm, 10 nm, and 20 nm) [7]. By analyzing over 35 microspheres under a 222 mT external magnetizing field, the peak-to-peak stray field amplitudes of 41 ± 1 μT for 5 nm and 10 nm superparamagnetic MNPs were measured, and these measurements reflect their comparable magnetic response. Finite-element simulations confirm variations in the MNP distribution and magnetization uniformity within the vaterite matrix. This high-resolution magnetic imaging approach yields critical insights into MNP-loaded vaterites, enabling their optimized synthesis and magnetically controlled systems for precision therapies and diagnostics.

The complex dielectric permittivity has been studied with waves of millimeter wavelengths for both rare earth manganate and titanate and for LiCoPO_4_ and LiNi_0.5_Co_0.5_PO_4_ orthophosphate composites. The measurements were performed in the 26 to 38 GHz range by determining the transmission and reflection coefficients through a plate [8]. The authors discuss the non-monotonic frequency dependence observed in both the real and imaginary components of the permittivity. In addition, the structural and phase compositions of the nanocomposites were examined to support the interpretation of the electromagnetic behavior.

Semiconductor bridges (SCBs), which represent a novel category of pyrotechnic devices, have garnered significant attention in both military and civilian sectors due to their distinctive operating principles and broad application potential. The complex burst characteristic parameters of SCBs were subjected to dimensionality reduction using principal component analysis (PCA), which enabled accurate evaluations of the output performance of SCBs. The accuracy and reliability of the PCA method were also validated. A 100 μF tantalum capacitor was used to excite the SCB, while a digital oscilloscope recorded the characteristic parameters of the SCB explosion [9]. The experimental results demonstrate that the critical burst time of the SCB decreases with the rising voltage, with the critical burst energy decreasing first and then increasing with the rising voltage. The total burst time and total burst energy of SCBs all decrease first and then increase with the rise in voltage. The SCB was utilized to ignite lead styphnate (LTNR) under varying circuit conditions, and the characteristic parameters obtained were analyzed using PCA to obtain comprehensive scores. The consistency between the two sets of scores validated the accuracy and reliability of PCA in assessing the SCB’s output capability.

Improving the energy release and safety of composite solid propellants is a key focus in energetic materials research. Graphene, with its excellent thermal conductivity and lubrication properties, is a promising additive. The propellants’ performance can be enhanced by graphene’s large surface area and excellent thermal and electrical conductivity, which promote AP decomposition and combustion, combined with its lubricating effect, which enhances safety. However, excessive graphene may hinder these benefits. The Al@AP core–shell particles doped with graphene were prepared via an in situ deposition method. The structure, thermal decomposition, combustion, and safety performance of the graphene-doped Al@AP samples were investigated [10]. The results showed that AP effectively coated the aluminum to form a typical core–shell structure, with graphene uniformly loaded into the framework. Closed-bomb and laser ignition tests revealed that the pressure rise rates and combustion intensity increased with the graphene content up to 1.0 wt.%, but beyond that they declined. Additionally, graphene significantly improved safety by reducing the sensitivity to impact and friction. This study provides balanced design criteria for graphene-doped Al@AP as a solid propellant.

Very-low-frequency (VLF, frequency range 3–30 kHz) radio waves play a crucial role in long-distance underwater navigation and communication due to their stable propagation characteristics and low attenuation rates. However, the applications of traditional VLF transmitter antennas (e.g., *T*-shaped antennas, umbrella antennas, etc.) are restricted by their wavelength limitations, their large physical dimensions (which usually reach several square kilometers), and the need to lay a large-area ground network. As a result, the construction of miniaturized VLF antennas is a current research hotspot. Research and experiments have been conducted to create some feasible solutions which promote the miniaturization of VLF antennas, such as mechanical antennas and magnetic resonant coupled antennas. In order to break through the difficulties of very-low-frequency (VLF) miniaturized antenna which have a small power capacity and low radiation efficiency, this paper proposes a high-radiation-field-strength magnetic loop antenna based on a nanocrystalline alloy magnetic core. A high-permeability nanocrystalline toroidal core (*μ_r_* = 50,000, *B_s_* = 1.2 T) is used to optimize the thickness-to-diameter ratio (t = 0.08) and to increase the effective permeability to 11,000. The Leeds wires, characterized by their substantial carrying capacity, are manufactured through a toroidal winding process [11].

Terahertz technology covers a broad spectrum of application prospects in a range of fields, such as communication, the military, and medicine, and the use of metamaterials is important for further developments of terahertz technology. Ning et al. [12] proposed a multi-mode switchable ultra-wideband terahertz absorber based on patterned graphene and VO_2_. They designed a graphene pattern which was composed of a large rectangle, rotated 45° in the center, and four identical small rectangles in the periphery, as well as a VO_2_ layer pattern composed of four identical rectangular boxes, with small rectangles embedded in the dielectric layer. The absorption mechanism was revealed by using the relative impedance theory and the electric field distribution, along with local plasma resonance analysis.

Prelithiation is widely considered one of the most promising strategies to compensate for the loss of active substances and to improve the initial Coulombic efficiency in silicon-based anodes for advanced high-energy-density batteries. However, because of their unstable solid electrolyte interface (SEI) layer and low initial Coulombic efficiency, they expand in volume during prelithiation and react with moisture, making commercialization a difficult process. A strategy using lithium bis(fluorosulfonyl)imide (LiFSI) treatment to eliminate redundant lithium was developed and LiF-based inorganic compounds on the surface of the prelithiated electrode were generated [13]. This technique not only increases the battery’s energy density but also its cycle stability, resulting in superior capacity retention and a longer cycling life.

Gallium oxide (Ga_2_O_3_), an ultra-wide bandgap semiconductor, is an ideal material for solar-blind photodetectors, but challenges, such as its low responsivity and response speed, persist. One-dimensional (1D) Ga_2_O_3_ nanorods were designed to achieve high photodetection performance [14]. Through modulating the source concentration, pH value, temperature, and reaction time, 1D *β*-Ga_2_O_3_ nanorods were controllably fabricated using a cost-effective hydrothermal method, followed by post-annealing. The findings highlight the potential of 1D Ga_2_O_3_ nanostructures to be used in high-performance solar-blind ultraviolet photodetectors, paving the way for future integrable deep ultraviolet optoelectronic devices.

With the continuation of industrialization, the problem of water resource pollution has become increasingly serious, and the mismatch between the supply of and demand for water resources in China has thus become more acute. Therefore, solving the problem of water pollution and improving the water ecological environment are particularly important to enhance the utilization rate of water resources. Chi et al. [15] synthesized graphene oxide (GO)/zinc oxide (ZnO)/silver (Ag) composite materials and investigated their photocatalytic degradation performance with ciprofloxacin (CIP) under visible light irradiation. The GO/ZnO/Ag composites, which had different ratios, were prepared via an impregnation and chemical reduction method and characterized using X-ray diffraction (XRD), scanning electron microscopy (SEM), Fourier transform infrared spectroscopy (FT-IR), and X-ray photoelectron spectroscopy (XPS). This catalyst effectively degraded ciprofloxacin under light irradiation, showing the potential for its use in water purification applications.
